# Serum hydroxycotinine was associated with chronic kidney disease (CKD): a cross-sectional study based on NHANES

**DOI:** 10.1080/0886022X.2024.2356024

**Published:** 2024-06-04

**Authors:** Meng’en Zhu, Zhimin Bi, Yaoling Wang, Wei Li

**Affiliations:** a Department of Geriatrics, Union Hospital, Tongji Medical College, Huazhong University of Science and Technology, Wuhan, China; b Department of Nephrology, Tongren Hospital of Wuhan University (Wuhan Third Hospital), Wuhan University, Wuhan, P.R. China

**Keywords:** NHANES, CKD, eGFR, smoke, serum hydroxycotinine

## Abstract

**Objective:**

Smoking has been suggested as a modifiable and cardiovascular risk factor for chronic kidney disease (CKD). Although long-term smoking has been associated with CKD, the potential relationship between its metabolite hydroxycotinine and CKD has not been clarified.

**Methods:**

A total of 8,544 participants aged 20 years and above from the National Health and Nutrition Examination Survey (NHANES) 2017 - March 2020 were enrolled in our study. CKD was defined by estimated glomerular filtration rate (eGFR) < 60 mL/(min*1.73 m^2^). Serum hydroxycotinine was measured by an isotope-dilution high-performance liquid chromatography/atmospheric pressure chemical ionization tandem mass spectrometric (ID HPLC-APCI MS/MS) method with a lower limit of detections (LLOD) at 0.015 ng/mL. The non-linear relationship was explored with restricted cubic splines (RCS). Pearson’s correlation coefficient and a multivariate logistic regression model were used for correlation analysis.

**Results:**

Serum hydroxycotinine and eGFR were negatively correlated in both non-CKD group (r= −0.05, *p* < 0.001) and CKD group (r= −0.04, *p* < 0.001). After serum hydoxycotinine dichotominzed with LLOD, serum hydroxycotinine ≥ 0.015 ng/mL was negatively correlated with eGFR not only in non-CKD group (r = −0.05, *p* < 0.001) but also in CKD group (r = −0.09, *p* < 0.001). After adjusting for comprehensive confounders, results from the multivariate logistic regression analysis showed that participants with serum hydroxycotinine ≥ 0.015 ng/mL had increased odds of CKD (OR = 1.505, *p* < 0.001).

**Conclusions:**

Serum hydroxycotinine might be positively associated with CKD. Further study is warranted to find the right concentration of hydroxycotinine to measure the CKD.

## Introduction

CKD affects 8% to 16% of global population and becomes one of the leading causes of death in developed countries [1]. CKD and its comorbidities impose an enormous economic burden on individual and society [2].

Smoking has been previously identified as a known exacerbator for a series of diseases, including CKD [3–8]. Many studies have showed that current and former smokers have higher risks for CKD than nonsmokers [9]. Although smoking and CKD are both associated with high rates of morbidity and mortality, the mechanisms involved are complex. Impairment of kidney function, caused by tobacco smoking, was thought to result from decreasing renal plasma flow and increasing albumin excretion. Tobacco smoking has been strongly confirmed to raise renal oxidative stress and kidney failure [10].

As is well-known, hydroxycotinine, one of the nicotinine metabolites measured in serum, was often considered as a superior biomarker for identifying tobacco exposure due to the higher blood concentration and longer residence time [11]. Vast majority of the absorbed nicotine is transformed to cotinine, and more than half of cotinine is further converted to hydroxycotinine. Indicating that hydroxycotinine is the primary metabolite of absorbed nicotine [11, 12]. Both physiological factors (age, gender, race, ethnicity, kidney function, genetics, etc.) and drug use could affect pharmacokinetics of nicotine, which leads to individual variation. Except in serum, cotinine and hydroxycotinine also could be quantified in a number of biological matrices, including urine, saliva, hair, etc. [11]. Compared to large fluctuations of hydroxycotinine concentration in urinary, it is more stable in serum, so the serum hydroxycotinine can be representative of the circulating levels [11, 13]. Lee J et al. [14] found that serum cotinine is associated with increased prevalence of CKD. Eid HA et al. [15] found that serum cotinine was significantly positive with tobacco exposure, the research had reinforced a conclusion that there was a negative correlation between serum cotinine and the eGFR. However, the association between serum hydroxycotinine, transformed by continine, and CKD is yet to be elucidated.

To fill the above research gap, we conducted a large cross-sectional study to investigate the potential association between serum hydroxycotinine and CKD based on the 2017 - March 2020 NHANES data. This will also provide evidence to emphasize the importance of smoking cessation in CKD prevention.

## Methods

### Data source

The NHANES is a well-established, cross-sectional survey representative of the entire American population. It is conducted by the National Center for Health Statustics of the U.S. Centers for Disease Control and Prevention to assess the nutiritional and physical status of non-institutionalized civilian residents in America, and consists of demorgraphic data, dietary data, examination data, qustionnaire data, etc [16]. Data used in this study were extracted from NHANES database (2017 to March 2020).

### Study population

**Figure 1.**
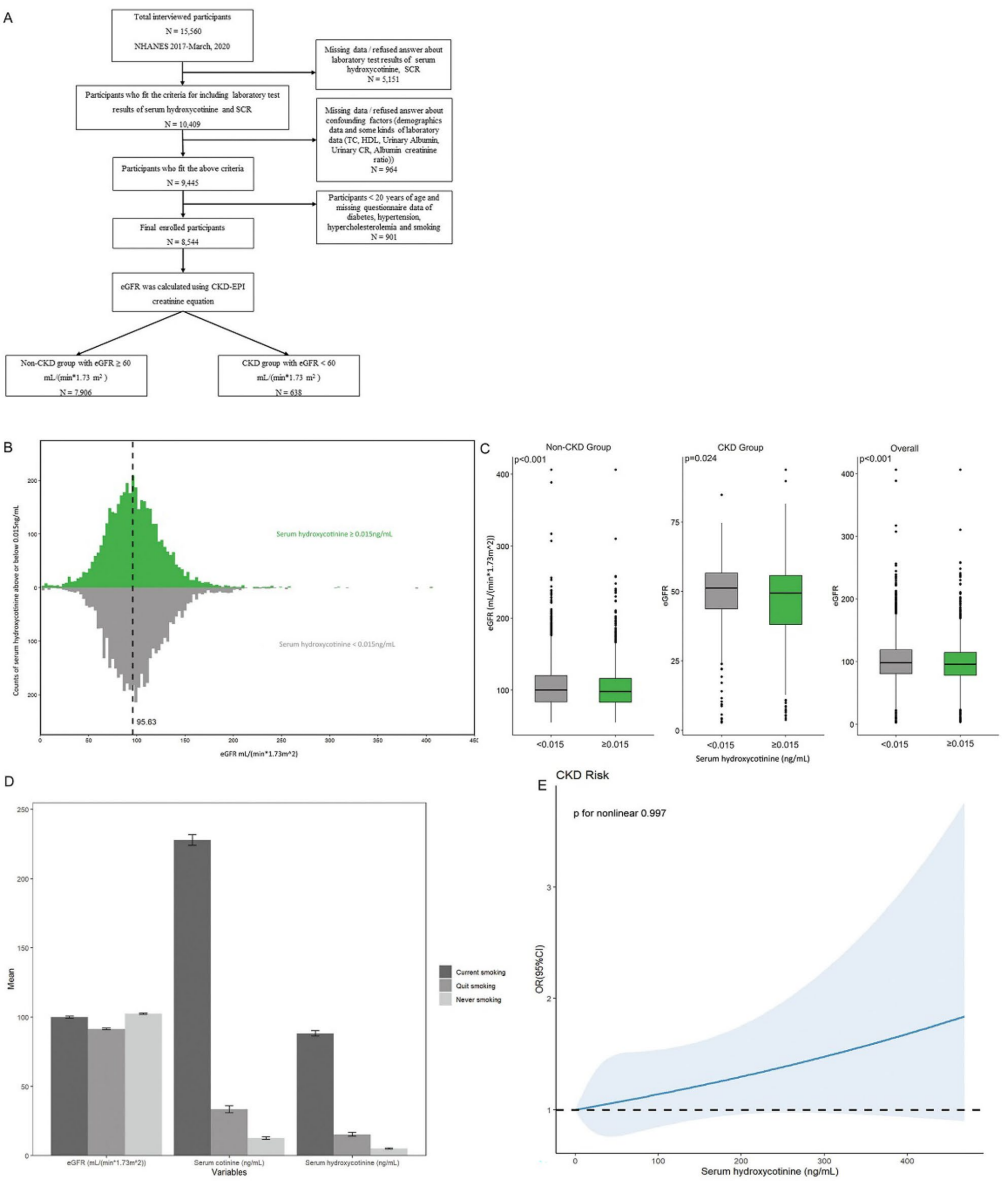
(A) Study population.Distribution (B) and distinction (C) of eGFR with serum hydroxycotinine categorized by LLOD at 0.015 ng/mL in baseline data. (D) Effects of different smoking status on eGFR, serum cotinine and serum hydroxycotinine using baseline data. (E) Restricted cubic spline models for the relationship between serum hydroxycotinine and the CKD using baseline data. The 95% CI of the adjusted OR was represented by the gray-shaded area. The model was adjusted for age, BMI, gender, race (Mexican American, other Hispanic, non-Hispanic white, non-Hispanic black, Non-Hispanic Asian, and other race), education (Less than 9th grade, 9-11th grade (Includes 12th grade with no diploma), High school graduate/GED or equivalent, Some college or AA degree, College graduate or above), marital status (married/living with partner, widow/divorced/separated, and never married), FPIR, hypertension, hypercholesterolemia, diabetes, smoking (current smoking, quit smoking, never smoking), TC, HDL, Urinary Albumin, Urinary CR, Albumin creatinine ratio. (CI, confidence interval; OR, odds ratio.).

We included 8,544 adult participants from NHANES 2017-March 2020 who met our study criteria. From a denominator of 15,560 total participants, we excluded 5,151 without data of serum hydroxycotinine concentration and standard biochemistry profile (including blood urea nitrogen (BUN), serum creatinine (SCR)) and additional 964 missing data/refused answer about confounding factors including demographics data and some kinds of laboratory data (including total cholesterol (TC), high-density lipoprotein (HDL), urinary albumin, urinary creatinine (CR) and albumin creatinine ratio), finally 901 aged under 20 years were also excluded because those paticipants had not yet proffered questionnaire data of hypertension, hypercholesterolemia, diabetes and smoking (Figure 1A).

### Definition of disease and covariates

CKD cases were defined as individuals with an eGFR < 60 mL/(min*1.73 m^2^) [1, 2] and eGFR was calculated using the CKD Epidemiology Collaboration (CKD-EPI) creatinine equation [17, 18]. The enrolled participants were divided into non-CKD group with eGFR ≥ 60 mL/(min*1.73 m^2^) and CKD group with eGFR < 60 mL/(min*1.73 m^2^). Finally, there were 7,906 participants enrolled in non-CKD group and 638 participants enrolled in CKD group (Figure 1A).

In our study, we also included the following covariates of interest which have been previously found to be associated with CKD [3]: age, body mass index (BMI), gender, race (Mexican American, other Hispanic, non-Hispanic white, non-Hispanic black, Non-Hispanic Asian, and other race), education (Less than 9th grade, 9-11th grade (Includes 12th grade with no diploma), High school graduate/General Educational Development (GED) or equivalent, Some college or Associate (AA) degree, College graduate or above), marital status (married/living with partner, widow/divorced/separated, and never married), family poverty income ratio (FPIR, categorized as ≤ 1, from 1 to 3, and > 3), history of diabetes, hypertension, hypercholesterolemia and smoking, serum cotinine, TC, HDL, urinary albumin, urinary CR, albumin creatinine ratio. Diabetes, hypertension and hypercholesterolemia were ascertained based on self-reports. Smoking status was categorized into current smoking (smoke every day or some days and had smoked at least 100 cigarettes in life time), quit smoking (currently not smoke but have smoked more than 100 cigarettes in the past) and never smoking (have smoked less than 100 cigarettes in lifetime) [19]. Serum cotinine and serum hydroxycotinine were measured by an isotope-dilution high-performance liquid chromatography/atmospheric pressure chemical ionization tandem mass spectrometric (ID HPLC-APCI MS/MS) method with a LLOD of 0.015 ng/mL [20].

### Statistical analysis

Comparisons of baseline characteristics were analyzed using Student’s t-test or nonparametric Mann-Whitney U test for continuous variables, as well as the Chi-square test or Fisher’s test for categorical variables. Serum cotinine and hydoxycotinine were dichotominzed at 0.015 ng/mL in our study. Histograms and boxplots were uesd to visually examine the distribution and distinction of eGFR stratified by LLOD of serum hydroxycotinine. Subgroup analysis was performed to further analyze, stratified by smoking status, and explore effects of tobacco smoking on eGFR, serum cotinine and serum hydroxycotinine. The correlation analysis between serum hydroxycotinine and baseline characteristics was calculated by Pearson’s correlation coefficient [21]. Propensity score matching (PSM) has become a popular statistical method for reducing selection bias in observational studies [22]. To explore the potential link between serum hydroxycotinine ≥ 0.015 ng/mL and CKD, we conducted PSM with a 1:1 ratio using the nearest neighbor matching algorithm to balance the baseline characteristics between the CKD group and non-CKD group and minimize potential confounding. We chose confounding factors (age, BMI, gender, race, education, marital status, FPIR, diabetes, hypertension, hypercholesterolemia, smoking, TC, HDL, Urinary Albumin, Urinary CR, Albumin creatinine ratio) for matching. RCS functions were effective tools to show a dose-response association between continuous exposures and outcomes [23]. We assessed the dose-response associations of serum hydroxycotinine with CKD using RCS to allow for potential non-linearity. Additionally, Logistic regression analysis was used to identify the risk factors that may threaten CKD. Differences were considered statistically significant at *p* < 0.05. All data analysis and figure design were performed by R studio (version 4.2.2).

## Results

### Participants’ baseline characteristics

A toal of 8,544 participants had data available for inclusion in our study. The participants age range was 20-80 years, and the mean ± SD was 50.99 ± 17.55 years. The BMI ± SD mean was 30.06 ± 7.59. About 51.6% of the population consisted of females, 34.3% were non-hispanic white, 32.4% had some college or AA degree, 57.6% were married/living with partner, and 43.2% of FPIR were range from 1 to 3. Diabetes was present in 15.5%, 38.6% were surffer from hypertension, and 35.7% had hypercholesterolemia. In addition, 58.3% of the population had never smoked. The baseline characteristics of the subjects are approved in Table 1. In the total subjects, excluding serum cotinine ≥ 0.015 ng/mL, serum cotinine and urinary CR, the other covariates were in connection with CKD (*p* ≤ 0.005). When serum hydroxycotinine was categorized by LLOD, the distribution and distinction of eGFR in CKD group and non-CKD group were presented in Figure 1B and C.

**Table 1.** Baseline characteristics for the non-CKD group and CKD group, as well as stratified by LLOD of serum hydroxycotinine at 0.015 ng/mL.

Characteristics	Non-CKD Group				CKD Group				Overall Subjects	*p*
	Serum Hydroxycotinine				Serum Hydroxycotinine				*N* = 8544	
	< 0.015ng/mL	≥ 0.015ng/mL	All		< 0.015ng/mL	≥ 0.015ng/mL	All			
	*N* = 4127	*N* = 3779	*N* = 7906	*p*	*N* = 282	*N* = 356	*N* = 638	*p*		
Age (y),(Mean (SD))	51.90 (17.22)	46.98 (16.72)	49.55 (17.16)	<0.001	70.65 (10.87)	67.51 (11.55)	68.90 (11.35)	<0.001	50.99 (17.55)	<0.001
BMI (kg/m^2^), (Mean (SD))	29.85 (7.05)	30.11 (8.08)	29.98 (7.56)	0.123	31.21 (6.87)	30.94 (8.49)	31.06 (7.81)	0.673	30.06 (7.59)	0.001
Gender (%)				<0.001				0.876		<0.001
Male	1805 (43.7)	1976 (52.3)	3781 (47.8)		155 (55.0)	199 (55.9)	354 (55.5)		4135 (48.4)	
Female	2322 (56.3)	1803 (47.7)	4125 (52.2)		127 (45.0)	157 (44.1)	284 (44.5)		4409 (51.6)	
Race (%)				<0.001				<0.001		<0.001
Mexican American	670 (16.2)	280 (7.4)	950 (12.0)		26 (9.2)	9 (2.5)	35 (5.5)		985 (11.5)	
Other Hispanic	518 (12.6)	330 (8.7)	848 (10.7)		22 (7.8)	20 (5.6)	42 (6.6)		890 (10.4)	
Non-Hispanic White	1360 (33.0)	1327 (35.1)	2687 (34.0)		123 (43.6)	122 (34.3)	245 (38.4)		2932 (34.3)	
Non-Hispanic Black	782 (18.9)	1248 (33.0)	2030 (25.7)		86 (30.5)	175 (49.2)	261 (40.9)		2291 (26.8)	
Non-Hispanic Asian	663 (16.1)	347 (9.2)	1010 (12.8)		15 (5.3)	12 (3.4)	27 (4.2)		1037 (12.1)	
Other Race - Including Multi-Racial	134 (3.2)	247 (6.5)	381 (4.8)		10 (3.5)	18 (5.1)	28 (4.4)		409 (4.8)	
Education (%)				<0.001				0.006		<0.001
Less than 9th grade	375 (9.1)	238 (6.3)	613 (7.7)		31 (10.9)	35 (9.8)	66 (10.4)		679 (8.0)	
9-11th grade (Includes 12th grade with no diploma)	328 (7.9)	530 (14.0)	858 (10.9)		27 (9.6)	62 (17.4)	89 (13.9)		947 (11.1)	
High school graduate/GED or equivalent	757 (18.3)	1129 (29.9)	1886 (23.9)		69 (24.5)	105 (29.5)	174 (27.3)		2060 (24.1)	
Some college or AA degree	1249 (30.3)	1322 (35.0)	2571 (32.5)		93 (33.0)	107 (30.1)	200 (31.3)		2771 (32.4)	
College graduate or above	1418 (34.4)	560 (14.8)	1978 (25.0)		62 (22.0)	47 (13.2)	109 (17.1)		2087 (24.4)	
Marital status (%)				<0.001				<0.001		<0.001
Married/Living with Partner	2699 (65.4)	1911 (50.6)	4610 (58.3)		172 (61.0)	137 (38.5)	309 (48.4)		4919 (57.6)	
Widowed/Divorced/Separated	792 (19.2)	890 (23.6)	1682 (21.3)		93 (33.0)	176 (49.4)	269 (42.2)		1951 (22.8)	
Never married	636 (15.4)	978 (25.8)	1614 (20.4)		17 (6.0)	43 (12.1)	60 (9.4)		1674 (19.6)	
FPIR (%)				<0.001				<0.001		<0.001
FPIR ≤ 1	537 (13.0)	1019 (27.0)	1556 (19.7)		42 (14.9)	101 (28.4)	143 (22.4)		1699 (19.9)	
1 < FPIR ≤ 3	1615 (39.1)	1767 (46.8)	3382 (42.8)		128 (45.4)	183 (51.4)	311 (48.7)		3693 (43.2)	
FPIR > 3	1975 (47.9)	993 (26.3)	2968 (37.5)		112 (39.7)	72 (20.2)	184 (28.8)		3152 (36.9)	
Diabetes (%)				0.018				0.019		<0.001
Yes	586 (14.2)	490 (13.0)	1076 (13.6)		103 (36.5)	145 (40.7)	248 (38.9)		1324 (15.5)	
No	3403 (82.5)	3198 (84.6)	6601 (83.5)		163 (57.8)	205 (57.6)	368 (57.7)		6969 (81.6)	
Unknown	138 (3.3)	91 (2.4)	229 (2.9)		16 (5.7)	6 (1.7)	22 (3.4)		251 (2.9)	
Hypertension (%)				0.982				0.963		<0.001
Yes	1468 (35.6)	1350 (35.7)	2818 (35.6)		213 (75.5)	271 (76.1)	484 (75.9)		3302 (38.6)	
No	2659 (64.4)	2429 (64.3)	5088 (64.4)		69 (24.5)	85 (23.9)	154 (24.1)		5242 (61.4)	
Hypercholesterolemia (%)				<0.001				0.022		<0.001
Yes	1561 (37.8)	1145 (30.3)	2706 (34.2)		164 (58.2)	177 (49.7)	341 (53.4)		3047 (35.7)	
No	2566 (62.2)	2634 (69.7)	5200 (65.8)		118 (41.8)	179 (50.3)	297 (46.6)		5497 (64.3)	
Smoking (%)				<0.001				<0.001		<0.001
Current smoking	44 (1.1)	1422 (37.6)	1466 (18.5)		3 (1.1)	84 (23.6)	87 (13.6)		1553 (18.2)	
Quit smoking	947 (22.9)	823 (21.8)	1770 (22.4)		109 (38.7)	129 (36.2)	238 (37.3)		2008 (23.5)	
Never smoking	3136 (76.0)	15334(40.6)	4670 (59.1)		170 (60.3)	143 (40.2)	313 (49.1)		4983 (58.3)	
Serum cotinine ≥ 0.015 ng/mL (%)				<0.001				<0.001		0.482
Yes	1515 (36.7)	3736 (98.9)	5251 (66.4)		89 (31.6)	344 (96,6)	433 (67.9)		5684 (66.5)	
No	2612 (63.3)	43 (1.1)	2655 (33.6)		193 (68.4)	12 (3.4)	205 (32.1)		2860 (33.5)	
TC (mmol/L), (median [IQR])	4.81 [4.16, 5.53]	4.68 [4.01, 5.40]	4.73 [4.09, 5.48]	<0.001	4.46 [3.79, 5.33]	4.45 [3.75, 5.30]	4.45 [3.78, 5.30]	0.927	4.73 [4.06, 5.46]	<0.001
HDL(mmol/L), (median [IQR])	1.34 [1.11, 1.63]	1.29 [1.09, 1.58]	1.32 [1.09, 1.60]	<0.001	1.24 [1.06, 1.50]	1.24 [1.03, 1.53]	1.24 [1.03, 1.52]	0.609	1.32 [1.09, 1.60]	<0.001
Urinary Albumin (mg/L), (Median [IQR])	8.10 [4.10, 16.30]	9.60 [5.00, 19.60]	8.80 [4.50, 17.90]	<0.001	22.40 [9.03, 105.50]	29.85 [10.35, 145.33]	27.50 [9.53, 123.08]	0.149	9.30 [4.70, 19.70]	<0.001
Urinary CR (mg/dL), (Median [IQR])	103.00 [59.00, 163.00]	127.00 [74.00, 194.00]	114.00 [65.00, 177.00]	<0.001	108.00 [64.75, 157.75]	124.50 [74.75, 176.25]	118.00 [71.00, 170.00]	0.059	114.00 [65.00, 176.00]	0.383
Albumin creatinine ratio (mg/g), (median [IQR])	7.36 [4.85, 13.88]	7.36 [4.76, 13.79]	7.36 [4.80, 13.83]	0.576	17.49 [8.38, 113.11]	22.70 [8.82, 150.45]	19.18 [8.71, 130.17]	0.343	7.70 [4.90, 15.42]	<0.001
BUN (mmol/L), (median [IQR])	5.00 [3.93, 6.07]	4.64 [3.93, 5.71]	5.00 [3.93, 6.07]	<0.001	8.57 [6.78, 10.71]	8.21 [6.43, 10.35]	8.21 [6.78, 10.71]	0.178	5.00 [3.93, 6.07]	<0.001
SCR (μmol/L), (median [IQR])	70.72 [60.11, 83.10]	75.14 [63.65, 87.52]	73.37 [61.88, 84.86]	<0.001	120.66 [109.62, 141.88]	124.64 [114.04, 156.47]	123.76 [112.27, 147.41]	0.005	75.14 [62.76, 89.28]	<0.001
eGFR (mL/(min*1.73m^2^)), (median [IQR])	100.57 [84.16, 120.49]	98.14 [83.07, 116.39]	99.58 [83.64, 118.51]	<0.001	51.29 [43.71, 56.56]	49.41 [38.15, 55.72]	50.41 [40.27, 56.03]	0.024	97.09 [79.17, 116.30]	<0.001
Serum cotinine (ng/mL), (median [IQR])	0.01 [0.01, 0.02]	15.20 [0.12, 213.00]	0.03 [0.01, 9.09]	<0.001	0.01 [0.01, 0.02]	0.39 [0.06, 158.00]	0.03 [0.01, 1.05]	<0.001	0.03 [0.01, 7.48]	0.987
Serum hydroxycotinine (ng/mL), (median [IQR])	0.01 [0.01, 0.01]	4.24 [0.04, 71.85]	0.01 [0.01, 2.51]	<0.001	0.01 [0.01, 0.01]	0.21 [0.03, 58.30]	0.02 [0.01, 0.49]	<0.001	0.01 [0.01, 2.14]	0.005

SD, standard deviation; IQR, interquartile range; LLOD, lower limit of detection; CKD, chronic kidney disease; BMI, body mass index; AA degree, Associate degree; GED, General Educational Development; FPIR, family poverty income ratio; TC, total cholesterol; HDL, high-density lipoprotein; BUN, blood urea nitrogen; SCR, serum creatinine; eGFR, estimated glomerular filtration rate.

### Effects of different smoking status on eGFR, serum cotinine and hydroxycotinine

To assess the effects of tobacco smoking on eGFR, serum cotinine and hydroxycotinine, we stratified the analysis by smoking status, and statistically significant differences were found (*p* < 0.001) (Figure 1D). The results gave an indication that the mean eGFR decreased across smoking status categories associated with increased tobacco exposure, with the highest eGFR among those who never smoking (mean ± SD was 102.33 ± 32.19 mL/(min*1.73m^2^)). Using serum hydroxycotinine and cotinine to verify self-reported smoking status, hydroxycotinine mean concentrations (ng/mL) were 88.24 (SD, 76.79), 15.3 (SD, 59.49) and 5.09 (SD, 37.98) of current, quit and never smokers, repectively. The trend of serum continine concentrations was remarkably similar to that of serum hydroxycotinine. Serum cotinine concentrations (ng/mL) were significantly higher in those who current smoking (mean ± SD was 227.96 ± 150.94) than those who quit (mean ± SD was 33.44 ± 111.61) or never (mean ± SD was 12.51 ± 69.98) smoking.

### Nonlinear relationship analysis

We performed RCS analysis to clarify the relationship between serum hydroxycotininea and CKD more clearly (Figure 1E). We found that the prevanlence of CKD increased with increasing serum hydroxycotinine and couldn’t show a nonlinear dose-reponse relationship (*p* for nonlinear = 0.997).

### Correlation between serum hydroxycotinine and baseline characteristics

**Figure 2.**
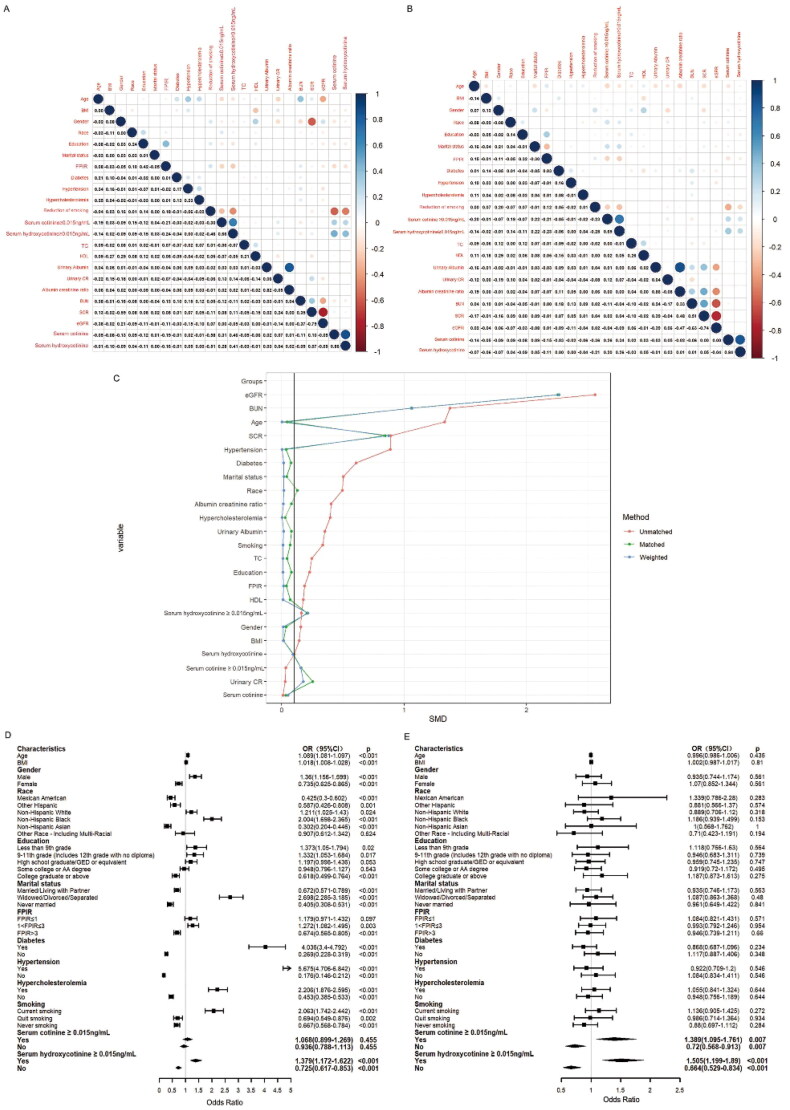
Correlation between serum hydroxycotinine ≥0.015 ng/mL and the baseline data in non-CKD group (A) and CKD group (B). The color represents the association’s strength. The Pearson’s correlation coefficient was used to calculate the correlation. (C) Distribution of SMD for the unmatched, matched and weighted non-CKD and CKD subjects with a ratio of 1:1. (SMD, Standardized mean difference). Forest plot of CKD risk factors before (D) and after (E) matching.

The associations of serum hydroxycotinine with baseline characteristics in non-CKD group (Figure 2A) and CKD group (Figure 2B) were evaluated by the Pearson’s correlation analysis. Serum hydroxycotinine had a negative correlation with eGFR in both non-CKD group and CKD group (r= −0.05, r= −0.04, *p* < 0.001, respectively). Serum hydroxycotinine ≥ 0.015 ng/mL was also negatively correlated with eGFR among participants with non-CKD (r= −0.05, *p* < 0.001) and CKD (r= −0.09, *p* < 0.001). In CKD group, the increase of eGFR was negatively correlated with diabetes (r= −0.09, *p* < 0.001), hypertension (r= −0.11, *p* < 0.001) and hypercholesterolemia (r= −0.04, *p* < 0.001), but it had a positive correlation with reduction of smoking (*r* = 0.02, *p* < 0.001). In CKD group, serum hydroxycotinine had a negative correlation with reduction of smoking (r= −0.21, *p* < 0.001), but it had a positive correlation with serum hydroxycotinine ≥ 0.015 ng/mL (*r* = 0.26, *p* < 0.001), serum cotinine (*r* = 0.84, *p* < 0.001) and serum cotinine ≥ 0.015 ng/mL (*r* = 0.20, *p* < 0.001). The similar outcomes were also found in non-CKD group. In non-CKD group, serum hydroxycotinine ≥ 0.015 ng/mL was negatively correlated with decreased smoking exposure (r = −0.46, *p* < 0.001), but positively correlated with serum cotinie ≥ 0.015 ng/mL (*r* = 0.66, *p* < 0.001), increased serum cotinie (*r* = 0.46, *p* < 0.001) and serum hydroxycotinine (*r* = 0.41, *p* < 0.001). The similar outcomes were also found in CKD group.

### Associations between factors and CKD before and after PSM

Figure 2D showed that multivariate logistic regression analysis was performed to analyze the associations between factors andCKD. Factors which had positive associations with CKD were age (OR= 1.089, *p* < 0.001), male (OR= 1.36, *p* < 0.001), subjects with current smoking status (OR = 2.063, *p* < 0.001), with marital status of widowed/divorced/separated (OR = 2.698, *p* < 0.001), with FPIR ranging from 1 to 3 (OR = 1.272, *p* = 0.003), diabetes (OR = 4.036, *p* < 0.001), hypertension (OR =5.675, *p* < 0.001), hypercholesterolemia (OR = 2.206, *p* < 0.001) and serum hydroxycotinine ≥ 0.015 ng/mL (OR= 1.379, *p* < 0.001). However, factors which had negative association with CKD were quit smoking (OR = 0.694, *p* = 0.002) and never smoking (OR = 0.667, *p* < 0.001). Most interestingly, the CKD with serum hydroxycotinine ≥ 0.015 ng/mL was 1.379 (*p* < 0.001), but serum hydroxycotinine < 0.015 ng/mL for CKD was 0.725 (*p* < 0.001).

Further on, to verify the association between serum hydroxycotinine ≥ 0.015 ng/mL and CKD, we create a comparable non-CKD group using the nearest neighbor PSM method (Figure 2C). In this study, all participants were divided into non-CKD group and CKD group. Before PSM, the two groups had significant differences in a major of covariates (*p* ≤ 0.005), but there was no difference in serum cotinine ≥ 0.015 ng/mL, serum cotinine and urinary CR (*p* > 0.05) (Table 2). 599 cases of non-CKD participants and 599 cases of CKD participants were successfully matched by PSM. After PSM, the characteristics of participants with and without CKD were similar, except for the following variables (including serum cotinine ≥ 0.015 ng/mL, serum hydroxycotinine ≥ 0.015 ng/mL, serum cotinine, serum hydroxycotinine, urinary albumin, urinary CR, albumin creatinine ratio, BUN, SCR and eGFR) (*p* < 0.05) (Table 2). After matching, non-CKD group and CKD group still had significant differences in serum hydroxycotinine (*p* = 0.002) and serum hydroxycotinine ≥ 0.015 ng/mL (*p* = 0.001). The results (Figure 2E) of the subsequent multivariate analysis showed that serum hydroxycotinine ≥ 0.015 ng/mL had increased odds of CKD (OR = 1.505, *p* < 0.001), but serum hydroxycotinine < 0.015 ng/mL had decreased odds of CKD (OR = 0.664, *p* < 0.001). Surprisingly, we also found that serum cotinine ≥ 0.015 ng/mL had significantly high odds of CKD (OR = 1.389, *p* = 0.007), and serum cotinine < 0.015 ng/mL had significantly low odds of CKD (OR = 0.72, *p* = 0.007).

**Table 2.** Characteristics of full and popensity score-matched cohorts by CKD.

	Before Matching				After Matching			
	Total Subjects	Non-CKD Group	CKD Group		Total Subjects	Non-CKD Group	CKD Group	
Characteristics	*N* = 8544	*N* = 7906	*N* = 638	*p*	*N* = 1198	*N* = 599	*N* = 599	*p*
Age (y),(Mean (SD))	50.99 (17.55)	49.55 (17.16)	68.90 (11.35)	<0.001	69.20 (10.95)	69.45 (10.55)	68.95 (11.34)	0.435
BMI (kg/m^2^), (Mean (SD))	30.06 (7.59)	29.98 (7.56)	31.06 (7.81)	0.001	31.02 (7.58)	30.97 (7.26)	31.07 (7.89)	0.81
Gender (%)				<0.001				0.6
Male	4135 (48.4)	3781 (47.8)	354 (55.5)		670 (55.9)	340 (56.8)	330 (55.1)	
Female	4409 (51.6)	4125 (52.2)	284 (44.5)		528 (44.1)	259 (43.2)	269 (44.9)	
Race (%)				<0.001				0.432
Mexican American	985 (11.5)	950 (12.0)	35 (5.5)		58 (4.8)	25 (4.2)	33 (5.5)	
Other Hispanic	890 (10.4)	848 (10.7)	42 (6.6)		85 (7.1)	45 (7.5)	40 (6.7)	
Non-Hispanic White	2932 (34.3)	2687 (34.0)	245 (38.4)		491 (41.0)	254 (42.4)	237 (39.6)	
Non-Hispanic Black	2291 (26.8)	2030 (25.7)	261 (40.9)		452 (37.7)	214 (35.7)	238 (39.7)	
Non-Hispanic Asian	1037 (12.1)	1010 (12.8)	27 (4.2)		50 (4.2)	25 (4.2)	25 (4.2)	
Other Race - Including Multi-Racial	409 (4.8)	381 (4.8)	28 (4.4)		62 (5.2)	36 (6.0)	26 (4.3)	
Education (%)				<0.001				0.775
Less than 9th grade	679 (8.0)	613 (7.7)	66 (10.4)		120 (10.0)	57 (9.5)	63 (10.5)	
9-11th grade (Includes 12th grade with no diploma)	947 (11.1)	858 (10.9)	89 (13.9)		168 (14.0)	86 (14.4)	82 (13.7)	
High school graduate/GED or equivalent	2060 (24.1)	1886 (23.9)	174 (27.3)		333 (27.8)	169 (28.2)	164 (27.4)	
Some college or AA degree	2771 (32.4)	2571 (32.5)	200 (31.3)		381 (31.8)	196 (32.7)	185 (30.9)	
College graduate or above	2087 (24.4)	1978 (25.0)	109 (17.1)		196 (16.4)	91 (15.2)	105 (17.5)	
Marital status (%)				<0.001				0.78
Married/Living with Partner	4919 (57.6)	4610 (58.3)	309 (48.4)		600 (50.1)	305 (50.9)	295 (49.2)	
Widowed/Divorced/Separated	1951 (22.8)	1682 (21.3)	269 (42.2)		488 (40.7)	238 (39.7)	250 (41.7)	
Never married	1674 (19.6)	1614 (20.4)	60 (9.4)		110 (9.2)	56 (9.3)	54 (9.0)	
FPIR (%)				<0.001				0.823
FPIR ≤ 1	1699 (19.9)	1556 (19.7)	143 (22.4)		252 (21.0)	122 (20.4)	130 (21.7)	
1 < FPIR ≤ 3	3693 (43.2)	3382 (42.8)	311 (48.7)		583 (48.7)	292 (48.7)	291 (48.6)	
FPIR > 3	3152 (36.9)	2968 (37.5)	184 (28.8)		363 (30.3)	185 (30.9)	178 (29.7)	
Diabetes (%)				<0.001				0.436
Yes	1324 (15.5)	1076 (13.6)	248 (38.9)		456 (38.1)	238 (39.7)	218 (36.4)	
No	6969 (81.6)	6601 (83.5)	368 (57.7)		704 (58.8)	344 (57.4)	360 (60.1)	
Unknown	251 (2.9)	229 (2.9)	22 (3.4)		38 (3.2)	17 (2.8)	21 (3.5)	
Hypertension (%)				<0.001				0.592
Yes	3302 (38.6)	2818 (35.6)	484 (75.9)		903 (75.4)	456 (76.1)	447 (74.6)	
No	5242 (61.4)	5088 (64.4)	154 (24.1)		295 (24.6)	143 (23.9)	152 (25.4)	
Hypercholesterolemia (%)				<0.001				0.686
Yes	3047 (35.7)	2706 (34.2)	341 (53.4)		628 (52.4)	310 (51.8)	318 (53.1)	
No	5497 (64.3)	5200 (65.8)	297 (46.6)		570 (47.6)	289 (48.2)	281 (46.9)	
Smoking (%)				<0.001				0.509
Current smoking	1553 (18.2)	1466 (18.5)	87 (13.6)		171 (14.3)	86 (14.4)	85 (14.2)	
Quit smoking	2008 (23.5)	1770 (22.4)	238 (37.3)		573 (47.8)	277 (46.2)	296 (49.4)	
Never smoking	4983 (58.3)	4670 (59.1)	313 (49.1)		454 (37.9)	236 (39.4)	218 (36.4)	
Serum cotinine ≥ 0.015 ng/mL (%)				0.482				0.008
Yes	5684 (66.5)	5251 (66.4)	433 (67.9)		771 (64.4)	363 (60.6)	408 (68.1)	
No	2860 (33.5)	2655 (33.6)	205 (32.1)		427 (35.6)	236 (39.4)	191 (31.9)	
Serum hydroxycotinine ≥ 0.015 ng/mL (%)				<0.001				0.001
Yes	4135 (48.4)	3779 (47.8)	356 (55.8)		609 (50.8)	274 (45.7)	335 (55.9)	
No	4409 (51.6)	4127 (52.2)	282 (44.2)		589 (49.2)	325 (54.3)	264 (44.1)	
TC (mmol/L), (median [IQR])	4.73 [4.06, 5.46]	4.73 [4.09, 5.48]	4.45 [3.78, 5.30]	<0.001	4.45 [3.78, 5.14]	4.40 [3.75, 5.04]	4.45 [3.78, 5.30]	0.379
HDL (mmol/L), (median [IQR])	1.32 [1.09, 1.60]	1.32 [1.09, 1.60]	1.24 [1.03, 1.52]	<0.001	1.24 [1.03, 1.50]	1.24 [1.03, 1.50]	1.24 [1.03, 1.53]	0.508
Urinary Albumin (mg/L), (Median [IQR])	9.30 [4.70, 19.70]	8.80 [4.50, 17.90]	27.50 [9.53, 123.08]	<0.001	16.80 [7.50, 61.52]	13.10 [6.50, 35.70]	23.20 [9.10, 92.95]	<0.001
Urinary CR (mg/dL), (Median [IQR])	114.00 [65.00, 176.00]	114.00 [65.00, 177.00]	118.00 [71.00, 170.00]	0.383	110.50 [67.00, 160.75]	103.00 [63.50, 149.00]	119.00 [71.00, 172.50]	<0.001
Albumin creatinine ratio (mg/g), (median [IQR])	7.70 [4.90, 15.42]	7.36 [4.80, 13.83]	19.18 [8.71, 130.17]	<0.001	14.75 [7.17, 58.48]	12.11 [6.77, 39.34]	17.72 [8.06, 90.93]	<0.001
BUN (mmol/L), (median [IQR])	5.00 [3.93, 6.07]	5.00 [3.93, 6.07]	8.21 [6.78, 10.71]	<0.001	6.78 [5.36, 8.93]	5.71 [4.64, 6.78]	8.21 [6.43, 10.53]	<0.001
SCR (μmol/L), (median [IQR])	75.14 [62.76, 89.28]	73.37 [61.88, 84.86]	123.76 [112.27, 147.41]	<0.001	100.78 [79.56, 121.99]	79.56 [68.07, 91.94]	121.99 [110.94, 142.32]	<0.001
eGFR (mL/(min*1.73m^2^)), (median [IQR])	97.09 [79.17, 116.30]	99.58 [83.64, 118.51]	50.41 [40.27, 56.03]	<0.001	61.25 [50.86, 86.24]	86.20 [72.68, 100.74]	50.85 [42.74, 56.21]	<0.001
Serum cotinine (ng/mL), (median [IQR])	0.03 [0.01, 7.48]	0.03 [0.01, 9.09]	0.03 [0.01, 1.05]	0.987	0.03 [0.01, 0.85]	0.02 [0.01, 0.53]	0.03 [0.01, 1.03]	0.014
Serum hydroxycotinine (ng/mL), (median [IQR])	0.01 [0.01, 2.14]	0.01 [0.01, 2.51]	0.02 [0.01, 0.49]	0.005	0.01 [0.01, 0.32]	0.01 [0.01, 0.19]	0.02 [0.01, 0.40]	0.002

SD, standard deviation; IQR, interquartile range; CKD, chronic kidney disease; BMI, body mass index; AA degree, Associate degree; GED, General Educational Development; FPIR, family poverty income ratio; TC, total cholesterol; HDL, high-density lipoprotein; BUN, blood urea nitrogen; SCR, serum creatinine; eGFR, estimated glomerular filtration rate.

## Discussion

This large cross-sectional study explored the potential relationship between serum hydroxycotinine and the CKD by merging and analyzing NHANES data from 2017 to March 2020. First of all, we also found that greater age, male gender, diabetes, hypertension, hypercholesterolemia, current smoking were risk factors for development of CKD [3]. Moreover, serum hydroxycotinine and cotinine were positively correlated, supporting that hydroxycotinine was a metabolite converted from cotinine. In addition, stratified analysis, differed by smoking status, revealed a negative relationship between increased serum concentration of hydroxycotinine and reduction of smoking, indicating that hydroxycotinine levels were consistent with tobacco exposure reduction. The conclusions of the present study were in accordance with previous findings [24]. The most important finding of this study was that serum hydroxycotinine had a negative correlation with eGFR, which suggested that increased serum concentration of hydroxycotinine could be associated with CKD. Further on, subjects with serum hydroxycotinine ≥ 0.015 ng/mL had significantly higher odds of CKD compared to those with serum hydroxycotinine < 0.015 ng/mL both before and after PSM.

To our knowledge, studies on serum hydroxycotinine and CKD are relatively limited. Hydroxycotinine was generally preffered as a primary biomarker of tobacco exposure because of its higher concentration and longer elimination half-lives [12, 13]. In present study, serum hydroxycotinine was negatively associated with reduction of smoking, as smoking status changing from current smoking to quit smoking to never smoking (*p* < 0.001). The association between smoking and incident CKD was described in a large number of studies [25]. Current and former smokers had higher risks of CKD than those who never smoked [25]. We also found that those who kicked the habit and nonsmokers had consistently lower risk of CKD. Exposure to tobacco smoke can be monitored by testing nicotine or its metabolites (cotinine and hydroxycotinine) in biological samples [10, 13]. Therefore, increased serum concentration of hydroxycotinine caused by tobacco smoking could directly act on cellular death pathways in renal cells, and the main mechanisms are redox system imbalance and inflammatory induction, thus lead to a decrease in renal function [26–28].

Hydroxycotinine, cotinine downstream metabolite, was postively correlated with cotinine. A negative correlation between serum cotinine and eGFR had been concluded, further emphasizing that as the smoking increased, it caused a decrease in renal function [15, 29]. Gu F et al. [12] identified that there was a does-dependent fashion between nicotine metabolites (cotinine, hydroxycotinine) and tobacco use. Lee J et al. [14] found that serum cotinine was associated with increased prevalence of CKD. In present study, we found a weak, but significant, negative correlation between serum cotinine and baseline eGFR among non-CKD subjects (r = −0.005, *p* < 0.001). After PSM, there was a significant difference in serum continie between non-CKD group and CKD group (*p* = 0.014). Those results reinforced the positive association between cotinine and increased prevalence of CKD. Our findings revealed that serum hydroxycotinine was negatively correlated with eGFR, supporting that increased serum hydroxycotinine might be associated with CKD. On basis of these results, we could suggest that serum hydroxycotinine could provid a warning of the adverse effects of tobacco smoke on CKD progression *via* cotinine. In addition, serum hydroxycotinine ≥ 0.015 ng/mL might be used as a valid clinical parameter because of its statistical significance with CKD (OR = 1.505, *p* < 0.001), but the exact serum concentration was uncertain.

However, our research still has some limitations. Firstly, in order to better relflect the relationship between different hydroxycotinine levels and CKD, we should set up more contrentration gradient groups (i.e. serum quartile groups). Secondly, the use of hydroxycotinine also has its limitations. Serum hydroxycotinine levels reflect tobacco exposure over the past few days [13], and those results might be misleading if the participants smoked occasionally or if the participants smoked less due to a period of illness. Thirdly, creatinine-based eGFR may not be an ideal marker for assessing the relationship between smoking and renal function [30]. Fourthly, while data had been adjusted for the potential confounders in this study, some potential residual confounders could still not be taken into account. Fifthly, around half of participants of 2017-2020 cycle were excluded due to missing personal data, which could seriously affect our research and therefore lead to widespread bias. Finally, more prospective, longitudinal cohort studies with multiple repetitive sampling are needed to help to support our observations.

## Conclusion

This is a large cross-sectional study to evaluate the association between serum hydroxycotinine and CKD, based on the NHANES database. Our study reinforced that serum hydroxycotinine was a key indicator for evaluating tobacco smoking. Additionally, serum hydroxycotinine had a negative correlation with eGFR, suggesting that serum hydroxycotinine might be positively associated with CKD. Further study is warranted to find the right concentration of hydroxycotinine to measure the CKD.

## Data Availability

The datasets presented in this study can be found in online repositories. The names of the repository/repositories and accession number(s) can be found below: www.cdc.gov/nchs/nhanes/
